# Assessment of Health-Related Quality of Life in Parkinson’s Disease: A Systematic Review

**DOI:** 10.7759/cureus.88061

**Published:** 2025-07-16

**Authors:** Oluwafolakemi M Aderinola, Kingsley O Ozojide, Ebere M Nwachukwu, Okelue E Okobi, Victoria Eneh, Jennifer C Mbonu, Oluchi C Abah, Toheeb Bakare, Emmanuel A Aniagbaoso, Gurinder Singh, Inemialu M Okhagbuzo

**Affiliations:** 1 Public Health, Nottingham Trent University, Nottingham, GBR; 2 Kinesiology: Exercise Science, Georgia Southern University, Statesboro, USA; 3 Family Medicine, Larkin Community Hospital Palm Springs Campus, Miami, USA; 4 Family Medicine, IMG Research Academy &amp; Consulting LLC, Homestead, USA; 5 Medicine, Ebonyi State University, Isieke, NGA; 6 Social Work, Hennepin Healthcare, Minneapolis, USA; 7 Medicine and Surgery, Crimean Federal University, Simferopol, RUS; 8 Family and Community Medicine, Abia State University, Uturu, NGA; 9 Internal Medicine/Neurology/Cardiology, Southmead Hospital, Bristol, GBR; 10 Internal Medicine, Enugu State University of Science and Technology College of Medicine, Enugu, NGA; 11 Family Medicine, China Three Gorges University, Yichang, CHN; 12 Internal Medicine, Babcock University, Ilishan, NGA

**Keywords:** health quality, health-related quality of life (hrqol), heath related quality of life, hrqol, parkinson's disease, parkinson’s disease (pd)

## Abstract

Different studies conducted on the effects of Parkinson’s Disease (PD) on the health-related quality of life (HRQoL) have presented contradictory outcomes, with the underlying domains of HRQoL requiring additional studies. Thus, the objective of this systematic review is to evaluate, by comparing and contrasting, the disease-specific HRQoL in PD. To attain the stated objective, a systematic review of various online databases, including PubMed, Scopus, Web of Science, Google Scholar, and MEDLINE, was conducted. Additionally, the study employed an increasingly robust methodology based on the Preferred Reporting Items for Systematic Reviews and Meta-Analyses (PRISMA) guidelines and Cochrane guidelines. Therefore, the study's inclusion criteria required that only studies published between 2010 and 2025, as well as those published in the English language, were to be included. The quality of included studies was further assessed via an appraisal tool for cross-sectional studies. For this study, a total of 10 studies satisfied the inclusion criteria and were subsequently reviewed. The included studies disclosed that PD patients had significantly poorer HRQoL compared to healthy and non-PD persons, driven by both the motor and non-motor systems (pain, fatigue, depression), medication effects, disease progression, and cognitive/social decline, with increased severity being observed in female and younger patients. Nevertheless, the psychiatric comorbidity data remain inconsistent. Thus, in PD, HRQoL is significantly impaired by the motor and non-motor symptoms, disease progression, and psychosocial aspects that require multidisciplinary care, improved assessment tools, as well as targeted interventions to tackle the mental and physical health for better patient outcomes.

## Introduction and background

Parkinson’s disease (PD) refers to a neurodegenerative disorder, mainly of the central nervous system, known to adversely affect the motor and non-motor systems [1-3]. Characteristic PD symptoms develop progressively, with non-motor symptoms becoming increasingly prevalent as the disease progresses. Among the notable motor symptoms are rigidity, postural reflex impairments, gait freezing, bradykinesia, and resting tremors; the non-motor symptoms have been acknowledged to develop at later stages of the disease and include neuropsychological dysfunctions that include depression, mood swings, psychosis, cognitive decline, fatigue, and sleep disturbances, all of which have been acknowledged to adversely affect the quality of life (QoL) of PD patients [2-7].

Despite a larger proportion of PD being idiopathic, various contributing factors have been acknowledged, with the disease’s pathophysiology involving progressive nerve cell degeneration within the substantia region of the midbrain, which secretes dopamine to the basal ganglia, which is the system tasked with voluntary motor control [3,8-13]. The hallmark degeneration of dopaminergic neurons in the substantia nigra disrupts basal ganglia function, leading to motor deficits, such as bradykinesia, tremor, rigidity, and postural instability, which compromise functional independence and the ability to perform daily activities [13]. However, growing evidence highlights the substantial impact of non-motor symptoms, including cognitive decline and emotional disturbances, on patient well-being [7]. Cognitive problems, often caused by inflammation in the brain and the buildup of alpha-synuclein, impact thinking skills, memory, and focus, while mood issues like depression and anxiety make it harder for patients to connect with others, cope emotionally, and stick to their treatment plans. Other possible causes of PD include environmental and genetic influences, as well as prior health conditions, medications, and lifestyle. New imaging methods like positron emission tomography (PET), magnetic resonance imaging (MRI), and dopamine transporter scan (DaTscan) have improved our ability to understand, detect early, and track both movement and non-movement symptoms of PD, leading to better management approaches.

The impact of PD on QoL varies significantly across global regions, influenced by socioeconomic factors, healthcare infrastructure, and cultural attitudes. In wealthy countries like South Korea and parts of Europe, getting an early diagnosis and having access to neurologists, rehabilitation services, and advanced treatments like deep brain stimulation help improve movement and overall care, but issues like REM sleep behavior disorder still negatively affect quality of life. Conversely, in low- and middle-income countries (LMICs), challenges such as delayed diagnosis, limited access to neurological care, medication shortages, and inadequate support systems exacerbate both motor and non-motor symptoms. For instance, in Saudi Arabia, restricted treatment options are associated with marked declines in QoL [14-17], while in Latin America, underrecognized non-motor symptoms, including depression, anxiety, and sleep disturbances, intensify the disease burden [1]. Cultural stigma in various regions also hinders disclosure and help-seeking behaviors, further limiting timely intervention [15]. These disparities point to the need for global health strategies that enhance awareness, diagnosis, and equitable access to comprehensive care for individuals with PD. Moreover, at present, the prevalence rate of PD stands at between 1 and 2 persons for every 1000 individuals [2]. Being a severe, progressive, and age-linked neurodegenerative disorder, PD is rare in younger individuals, but with a prevalence that may reach approximately 4% in older individuals [2].

Consequently, the World Health Organization (WHO) has described the health-related QoL (HRQoL) as the individual’s subjective perception of overall well-being, which encompasses their social, physical, mental, and emotional functioning, as influenced by their health status [4]. HRQoL not only considers physical health but also accounts for how the disease and treatment affect the patient’s daily life and his/her ability to live a fulfilling life in relation to his/her expectations, concerns, goals, and standards [4]. As such, HRQoL encompasses psychological, physical, cognitive, autonomy, environmental, and social relations factors. To enhance the HRQoL of PD patients, one must comprehend how the divergent domains of QoL deviate in PD patients and healthy individuals. Several comparative studies have been conducted on HRQoL in PD patients; however, the findings have been inconsistent, particularly concerning the degree of dissimilarities between PD patients and other individuals in diverse domains. Notably, several studies have disclosed that PD patients presented an overall lower HRQoL [5-7], even as additional studies have not disclosed any significant deviations in the HRQoL domains of mental health, environment, physical health, social relations, and emotional function [6]. In PD patients, the key correlates of HRQoL often include the comorbid depressive symptoms, as well as the PD subtypes and severity [8]. Among the notable factors that contribute to poor HRQoL outcomes are adverse medication effects, gait impairments, and psychosocial dysfunctions [3]. The key objective of this study is to assess and subsequently synthesize existing literature on the HRQoL in PD patients by comparing and contrasting the disease-specific HRQoL in PD.

## Review

Materials and methods

For this systematic review, we conducted an extensive literature search on different online databases, including PubMed, Web of Science, Scopus, Google Scholar, and Embase, for literature/studies published between 2010 and 2025. Thus, the studies selected for inclusion in this review included health assessment studies, epidemiological studies, systematic reviews, prospective cohort studies, and multicenter studies. Identification of duplicate data sources was conducted through a comparison of studies from similar population years. Additionally, various MeSH keywords were employed in the literature search, including Parkinson’s disease, quality of life, health status indicators, systematic review, and patient-reported outcome measures. The literature search conducted resulted in a total of 256 articles.

Inclusion and exclusion criteria

Following the removal of all duplicate studies, a selection of relevant studies was carried out based on a three-phase process. The initial phase entailed the screening of the titles and abstracts of the studies, whereas the second phase involved the exclusion of articles considered irrelevant to the study. Still, the third phase involved the performance of a comprehensive full-text evaluation of every reference to verify its significance. Therefore, three independent reviewers were tasked with screening the references, and potential disagreements were mainly resolved through discussions and consensus.

For this systematic review, the inclusion criteria mainly targeted original studies, including prospective cohort studies, randomized controlled trials (RCTs), and crossover studies, among others that met the following criteria: original scientific studies published in reliable and peer-reviewed journals, studies published between 2010 and 2025, full-text articles, and studies originally published in the English language. Additionally, to be included, the studies had to focus on assessing the health-related quality of life in PD. On the other hand, the exclusion criteria included expert opinion pieces, editorials, narrative reviews, sponsored clinical trials, and studies without relevance to the target populations. Further, inaccessible and irrelevant studies, along with studies with illogical methodologies, were excluded, resulting in the removal of 244 articles. Therefore, for this study, the data extracted from the included studies included general study attributes, including sampling methods, publication years, and authors; demographic attributes, including the sample size, sex, age, ethnicity/race, and follow-ups; and data pertaining to interventions and their duration. The key findings of each study were systematically recorded.

Results

**Figure 1:**
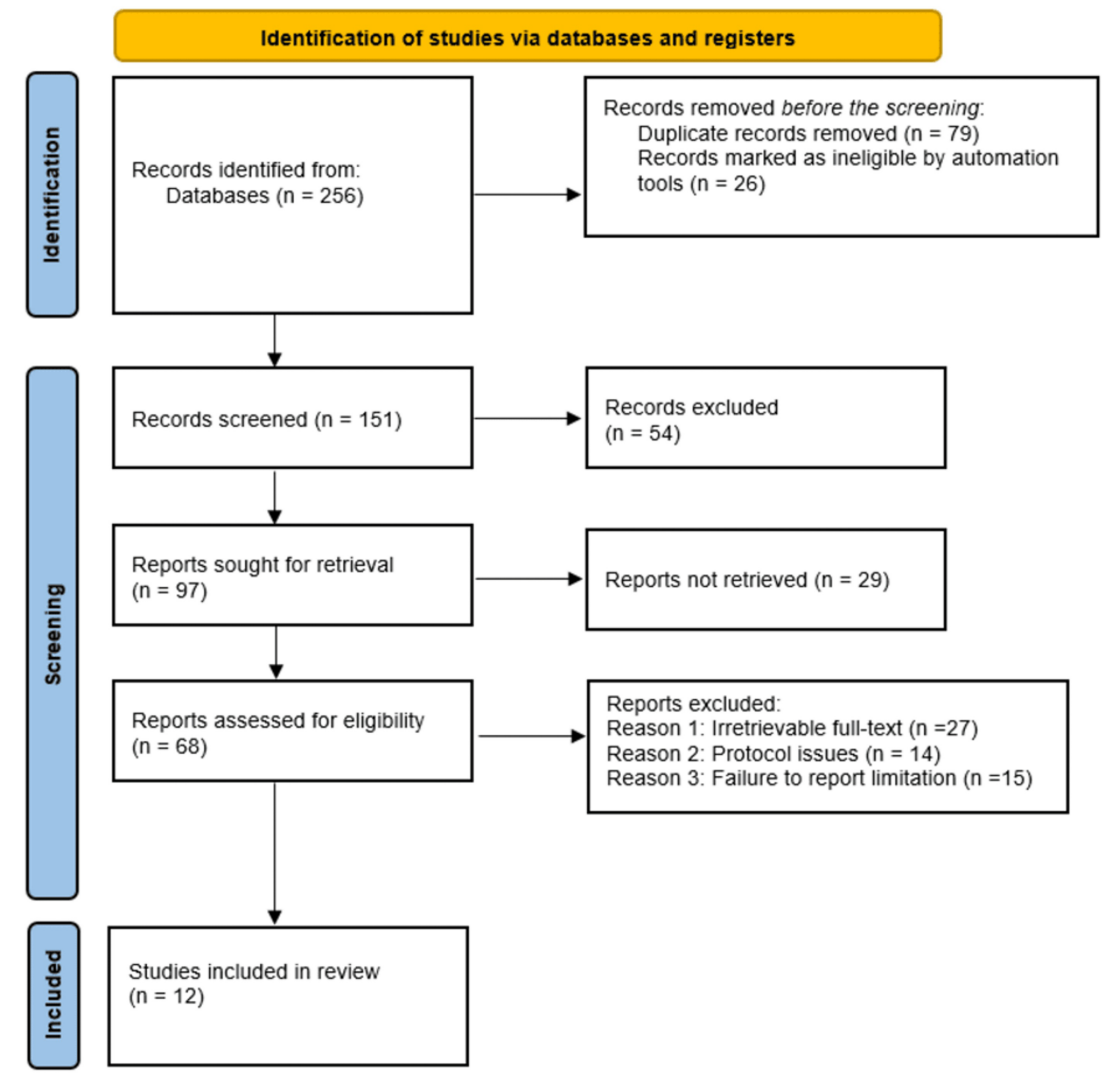
PRISMA flow diagram indicating the literature search and inclusion process for this systematic review. PRISMA: Preferred Reporting Items for Systematic Reviews and Meta-Analyses; n: Number

For this study, the Preferred Reporting Items for Systematic Reviews and Meta-Analyses (PRISMA) guidelines were used in the literature selection and inclusion, with the first exhaustive database search yielding 256 studies. After screening, 79 duplicates were excluded, and was followed by screening the studies’ titles and abstracts, which resulted in the automatic exclusion of 26 ineligible studies. The remaining 151 studies were further screened, leading to the exclusion of an additional 54 studies that were found ineligible. As a result, 97 studies were sought for retrieval, and 29 were found to be irretrievable. The remaining 68 studies were assessed for eligibility, and 56 studies were excluded for various reasons, including irretrievable full texts (27 studies), being protocols (14 studies), and failing to report limitations (15 studies). Ultimately, 12 studies met the inclusion criteria and were included in this systematic review. The included studies have been assessed and discussed jointly with the findings of other studies that corroborate our findings [5-19]. The PRISMA flow diagram outlining the article selection process for this study is shown in Figure 1.

Quality assessment

The quality of included studies was evaluated using AXIS, a 20-item critical appraisal tool for cross-sectional studies [9]. Thus, three independent reviewers were tasked with evaluating each study and resolving potential disagreements through consensus and discussion. The responses were also scored as 1 (yes), 0 (no), or “don’t know” for inapplicable items. Generally, most of the included studies were of high quality, with only three rated as moderate. Table 1 shows the results of the quality assessment of the included studies using the AXIS critical appraisal tool.

**Table 1:** AXIS quality assessment results for the included studies in this systematic review

Study/Citation	Clear aims	Design appropriate	Sample representativeness	Valid recruitment	Sample justified	Precision reported	Ethics approval	Consent	Outcome measures valid	Statistical methods appropriate	Results are reported	Discussion is balanced	Funding disclosed	Conflicts disclosed	Total score (/20)
Park et al. [5]	1	1	1	1	1	1	1	1	1	1	1	1	1	1	14
Hendred and Foster [7]	1	1	1	1	1	1	1	1	1	1	1	1	1	1	14
Lubomski et al. [8]	1	1	1	1	1	1	1	1	1	1	1	1	1	1	14
Ben-Shlomo et al. [10]	1	1	1	1	1	1	1	1	1	1	1	1	1	1	14
Chen [12]	1	1	1	1	1	1	1	1	1	1	1	1	1	1	14
Gazibara et al. [13]	1	1	1	1	1	1	1	1	1	1	1	1	1	1	14
Duncan et al. [14]	1	1	1	1	1	1	1	1	1	1	1	1	1	1	14
Santos García et al. [15]	1	1	1	1	1	1	1	1	1	1	1	1	1	1	14
Prasuhn et al. [16]	1	1	1	1	1	1	1	1	1	1	1	1	1	1	14
Al-Khammash et al. [17]	1	1	1	1	1	1	1	1	1	1	1	1	1	1	14
Tu et al. [18]	1	1	1	1	1	1	1	1	1	1	1	1	1	1	14
Heimrich et al. [19]	1	1	1	1	1	1	1	1	1	1	1	1	1	1	14

Table 2 shows a comparative analysis of the studies included in this systematic review.

**Table 2:** A comparative analysis of all the studies included in this systematic review EQ-5D: EuroQol 5-Dimension questionnaire; HRQoL: Health-Related Quality of Life; n: Number; N/A: Not Applicable; NMS: Non-Motor Symptoms; PD: Parkinson’s Disease; PDQ-8: Parkinson's Disease Questionnaire-8; PDQ-39: Parkinson's Disease Questionnaire-39; RBD: REM Sleep Behavior Disorder; REM: Rapid Eye Movement sleep; SF-36: 36-Item Short Form Health Survey; STN-DBS: Subthalamic Nucleus Deep Brain Stimulation; WHOQOL - Brief version: World Health Organization Quality of Life - BREF

Study/Citation	Study objective	Sample size (n)	Key findings	HRQoL assessment tool
Park et al. [5]	The study assessed the impact of possible RBD on HRQoL in PD patients with STN-DBS.	87	The study disclosed that probable RBD worsened HRQoL in PD patients with STN-DBS.	PDQ-39
Hendred and Foster [7]	The study assessed the WHOQOL-BREF in mild-to-moderate PD.	50	WHOQOL-BREF is valid for assessing HRQoL in mild-to-moderate PD	WHOQOL-BREF
Lubomski et al. [8]	The study assessed HRQoL in PD patients and caregivers	100 PD patients and 100 caregivers	The study disclosed that caregiver burden had a negative impact on the PD patients' HRQoL.	PDQ-39, EQ-5D
Ben-Shlomo et al. [10]	The study conducted an epidemiological review of PD.	Review (no specific n)	The study highlighted the global burden and economic impact of PD, and the effects on the quality of life of PD patients.	N/A
Chen [12]	The study evaluated the economic and HRQoL impacts of early PD treatment on the patients.	Review (no specific n)	The study disclosed that early treatment enhances HRQoL and reduces the long-term costs.	N/A
Gazibara et al. [13]	The study assessed the HRQoL in relation to the fall risk in PD patients.	120	The study disclosed that falls significantly worsened HRQoL in PD patients.	PDQ-39
Duncan et al. [14]	The study evaluated the impact of non-motor symptoms (NMS) on HRQoL in early PD patients.	159	The study disclosed that NMS, including depression and fatigue, strongly diminished HRQoL in early PD patients.	PDQ-39
Santos García et al. [15]	The study assessed the various predictors of significant HRQoL impairment in PD patients.	409	The study disclosed that motor severity, sleep disturbances, and depression were predictive of poor HRQoL.	PDQ-8
Prasuhn et al. [16]	The study evaluated NMS and HRQoL in mild Parkinson's disease.	715	Mild PD symptoms already reduce HRQoL	PDQ-39
Al-Khammash et al. [17]	The cross-sectional study assessed the HRQoL in PD.	100	Motor and non-motor symptoms equally affect HRQoL	PDQ-39
Tu et al. [18]	The study assessed the various determinants of generic and PD-specific HRQoL.	150	Non-motor symptoms are more strongly linked to PD-specific HRQoL	SF-36, PDQ-39
Heimrich et al. [19]	The researchers conducted a network analysis of NMS’ effects on HRQoL.	1,200	The study disclosed that depression and fatigue are the main NMS affecting HRQoL in PD patients.	PDQ-39

Data extraction

The authors used a data extraction form to extract pertinent data from included studies. Thus, data pertaining to the various attributes of the studies, including the general attributes of the studies, author names, and year of publication; demographic attributes, such as sample size, gender, age, follow-ups, and race; and the interventions used, intervention duration, and measurement methods were extracted. The systematic recording of the key study findings followed this.

Discussion

As a multidimensional concept, HRQoL reflects the subjective assessment of an individual’s fulfillment with life, and takes in aspects that include relationships with family and relatives, an individual’s health, finances, the close persons’ health, independence, housing, religion, leisure activities, and social life [10,11]. Health significantly contributes to the QoL, and the domain has been referred to as HRQoL. According to the WHO, health implies a state of complete physical, social, spiritual, and mental well-being, and not just the absence of infirmity or disease [4]. This description is indicative that social and psychological aspects are important parts of health. Though “role functioning” has regularly been added as a distinct entity to the HRQoL concept, Zhao et al. have taken into consideration various HRQoL descriptions and subsequently defined the concept as the optimal level of physical, mental, social, and role functioning, including aspects of health perceptions, relationship, life satisfaction, fitness, and well-being [11].

The assessment of HRQoL is mainly done using generic and disease-specific instruments, with the generic instruments including the Medical Outcomes Study-short form 36 sickness impact profile, which enables the comparison of HRQoL across diverse diseases [11]. Such instruments comprise items of increasingly general nature, and, as a result, lack specificity. Nevertheless, in PD, numerous disease-specific HRQoL instruments are available, even as investigators are faced with an increasing choice of different scales, which differ in several aspects.

According to Hendred and Foster, the HRQoL is mainly established through the overall balance between the distressing and protective factors [7]. Thus, the HRQoL is considered lower in instances where the distressing factors predominate over the protective factors. The psychosocial and motor functions, as well as psychiatric comorbidities, including cognitive decline, sleep disturbances, gait freezing, fatigue, depression, and bradykinesia, are widespread in PD patients and have been acknowledged to lower the patients’ quality of life. Specific demographics, including gender, age, education level, knowledge and beliefs, marital status, and living conditions, as well as clinical attributes that include duration of illness, disease severity, disease stage, and subtype, were found to be significantly linked to HRQoL in PD patients.

As expected of any chronic and gradually worsening condition, PD presents considerable impacts on the patients’ HRQoL. In a recent study, Chen reported that PD patients displayed lower scores concerning mental and physical HRQoL dimensions than patients with eight other chronic and neurological diseases, including congestive heart failure, stroke, diabetes, and angina/coronary heart disease [12]. Notably, the study disclosed that non-motor disability, especially insomnia, depression, alongside various mental health factors, had significant negative effects on HRQoL as compared to motor deficits [12].

Still, PD patients have been reported to have significantly poorer HRQoL in comparison to non-PD patients, with lower mental and physical scores, as well as greater perceived decline in health status [8]. Such declines correlate with the severity of the disease, including motor impairment, higher medication doses, depression, levodopa complications, reduced activity, and non-motor symptoms. The HRQoL in PD patients is further affected and worsened by factors such as unemployment, dependence on social services, and retirement, stressing the requirement for strong support systems [8]. Impulse control disorders (ICD) and REM sleep behavior disorder have additionally been found to adversely affect HRQoL as a result of psychiatric and sleep disturbances [5]. Advanced PD severity, including worsening motor symptoms, longer durations, motor complications, and higher medication requirements, also diminishes HRQoL [5,8].

Approximately 60% to 80% of PD patients are affected by gastrointestinal dysfunction, including bloating and constipation, and this has been associated with poorer HRQoL outcomes and increased motor burden, highlighting the requirement for improved GI management [8]. HRQoL was strongly worsened by depression, with a potential bidirectional correlation-mood impacting the perception of symptoms and vice versa. Nevertheless, a dual approach that targets the mood and other symptoms might enhance the outcomes, even as additional research is required to assess such correlations.

In a study that assessed the capability of diverse domains of HRQoL to predict falls in PD patients over 12 months, Gazibara et al. reported that PD patients who experienced falls had poorer HRQoL [13]. Fallers had significantly poor performance in all HQROL domains assessed using SF-36, except for the emotional domain role [13]. Such observations corroborate the findings of the Polish study that disclosed that every HRQoL domain evaluated using PDQ-39 was significantly lower in fallers than non-fallers with PD [13]. Additionally, a recent longitudinal study disclosed that PD patients experienced significant HRQoL declines over two years, mainly as a result of the worsening motor functions and the subsequent increment in non-motor symptom (NMS) burden [14]. Thus, higher NMS burden and mood impairment independently projected clinically significant HRQoL deterioration, impacting nearly 20% of PD patients, with younger persons and women being more vulnerable. Worsening NMS, including fatigue, cognitive issues, and pain, as well as motor decline, have also been reported in PD patients but not in healthy and non-PD patients, corroborating the findings of earlier studies [14]. Though more than half of PD patients were reported to have higher Parkinson's disease questionnaire 39 summary index (PDQ-39SI) scores at the time of follow-up, only about 19% satisfied the set threshold for clinically significant HRQoL decline (a ≥10% PDQ-39SI increase) [14,15]. Variations in rates of impairment across studies might reflect divergent HRQoL definitions. Regardless of the shorter follow-up duration, PD patients demonstrate a significant decline in HRQoL, even though the generic measures of quality of life are unlikely to effectively detect such changes.

Further, various studies have presented conflicting findings concerning the correlations between psychiatric comorbidities and HRQoL in PD patients. For instance, though some studies have indicated that depression was the strongest factor contributing to lower HRQoL in PD patients [3], not all [8,16]. Thus, pain, anxiety, and apathy have also been linked to poor HRQoL in PD patients and have been noted to have greater effect sizes compared to motor symptoms [7,17]. The correlations between anxiety and poor HRQoL in PD remain inconsistent between studies, as certain studies have reported no significant correlations [11]. Additionally, the findings on the correlations between sleep disturbance and HRQoL in PD are varied, even as certain studies have reported a correlation [5] while others have not [8,11]. Still, rapid eye movement (REM) sleep behavioral disorder and reductions in the levels of striatal dopamine transporter, as well as increments in PD-associated brain activities, might be linked to PD development [5]. Such discrepancies might be attributed to factors that include variations in sampling methods, assessment tools, severity of the disease, cognitive functions, genetic factors, and treatment effects [10]. Also, the limited number of studies that have utilized similar HRQoL measurements is likely to prevent the assessment of how such clinical and demographic factors impact HRQoL in PD.

Studies using SF-36 and PDQ-39 measurements have disclosed a significant correlation between depression and poorer physical and psychosocial functioning in PD patients [18]. Thus, the studies have shown that depression significantly affects multiple PDQ-39 domains, including emotional well-being, mobility, communication, and stigma [11]. Additionally, depression has been found to worsen everyday functioning, which further diminishes HRQoL [11]. Such findings have underscored the correlations between HRQoL, depression, and physical limitations, highlighting the significance of integrated care in the management of physical and psychological aspects of PD.

Consequently, fatigue has been reported to significantly lower HRQoL in PD, especially through its effects on mobility, and needs to be tackled regardless of the limited treatment alternatives [19]. The management of depression, which significantly affected HRQoL, particularly through its adverse effects on emotional well-being, might be important, given the overlap between depression and fatigue. Still, hyperhidrosis or excessive sweating mainly influences bodily discomfort, in addition to affecting all HRQoL domains, which makes it a valuable screening tool for assessing autonomic dysfunction [19]. Concentration challenges and excessive daytime sleepiness strongly affect cognition, in addition to acting as bridge symptoms, thereby worsening HRQoL in PD patients across all dimensions [19,20]. Such findings have emphasized the requirement for effective assessment and management of such non-motor symptoms to enhance the HRQoL and overall well-being of PD patients.

Younger-onset Parkinson's disease (YOPD), typically diagnosed between the ages of 21 and 40 years, presents distinct clinical and psychosocial challenges compared to late-onset Parkinson’s disease (LOPD) [21]. Individuals with YOPD are generally more physically active and cognitively preserved in the early stages of the disease. However, the earlier onset often results in a prolonged disease course with profound implications for personal, familial, social, and occupational functioning [21]. Unlike the LOPD population, which largely consists of retirees, those with YOPD are often in the midst of their most productive years. Many are primary income earners, young parents, or pregnant individuals-circumstances that compound the psychological burden of the diagnosis. This demographic frequently encounters elevated levels of psychosocial stress, which can lead to anxiety, depression, reduced household income, and diminished workforce participation, ultimately impacting community productivity. Furthermore, research indicates that motor complications associated with long-term levodopa therapy, including early and persistent dystonias, tend to manifest earlier and more frequently in YOPD patients [22]. Despite these challenges, individuals with YOPD often demonstrate significant resilience and adaptability and may benefit from stronger social support networks due to their younger age.

Deep brain stimulation (DBS) remains the most widely utilized device-assisted intervention for patients with moderately advanced PD experiencing motor complications, such as dyskinesias, although it is employed by only a minority of eligible individuals [23]. DBS has been shown to improve quality of life by reducing levodopa-induced dyskinesias, lowering the required dosage of dopaminergic medications, thereby minimizing side effects, and significantly decreasing medication-refractory tremors, which enhances patients’ ability to perform daily tasks such as eating and writing [23]. Pharmacological therapy continues to serve as the cornerstone of motor symptom management in PD, improving mobility by reducing rigidity and enhancing gait and balance, while also alleviating non-motor symptoms such as sleep disturbances and constipation. Complementary approaches, such as psychotherapy, address the emotional burden of chronic disease by reducing stress and supporting patients through grief and functional decline via mindfulness and counseling strategies. Additionally, regular exercise plays a critical role in maintaining physical function, increasing stamina and muscle strength, and further reducing constipation when combined with adequate hydration.

The multifaceted nature of PD and its impact on the social determinants of health (SDOH) necessitates comprehensive and systems-level responses. Some of the integrated and evidence-based strategies proposed for implementation by the government and hospitals to improve the HRQoL of PD patients have been discussed. First, regular screening programs for NMS are recommended, given that the non-motor symptoms, including anxiety, depression, sensory neuropathy, cognitive dysfunction, and sleep disturbances, are among the key predictors of poor HRQoL in PD patients [1,3,14]. Obligatory NMS screening would necessitate that every neurology and movement disorder clinic utilize validated tools, including the Non-Motor Symptoms Questionnaire (NMSQuest) and the PDQ-39 in the course of regular assessment appointments. Additionally, hospitals should implement policies, including annual mental health check-ins for NMS symptoms, including depression, anxiety, and sleep disorder screenings, for PD patients, particularly targeting conditions such as REM sleep behavior disorder, which adversely affects HRQoL [5]. Still, primary care provider (PCP) training, including government-funded workshops and online training modules, is essential to ensuring that the PCPs can recognize early non-motor signs and symptoms, and make apt referrals [2], which will assist in improving care and HRQoL for PD patients.

Second, the integration of mental health services into Parkinson’s care has been recommended, given the observation that mental health disorders, especially depression and anxiety, are increasingly prevalent despite being undertreated in PD patients [1,14]. Therefore, an effective approach includes establishing a collaborative care model that will integrate the services of mental health practitioners to offer on-site mental health consultations, evaluations, and interventions within the PD clinics' contexts during patient visits. However, for PD patients in rural and underserved regions, there is a need for government health departments to improve access to tele-mental health services through funding of telepsychiatry and telepsychology services. Furthermore, hospitals should run PD-specific group-based psychosocial programs, including PD-specific group cognitive behavioral therapy (CBT) and mindfulness-based stress reduction (MBSR) programs [8].

Thirdly, in recommending the implementation of community-based rehabilitation and fall prevention programs, it has been noted that falls and mobility-related issues increase hospitalization rates and contribute to poor HRQoL [13]. Therefore, home safety assessments should be conducted through government-funded partnerships between hospitals and occupational therapists as a means of preventing potential falls in PD patients. Moreover, it is recommended that municipalities should invest in accessible PD-specific exercise programs that include Tai Chi, balance training, and physiotherapy classes, which should be offered at the community centers. Still, it is recommended that hospitals establish specialized gait and balance clinics that offer personalized gait assessments, physical therapy, and assistive device training.

Fourth, multidisciplinary care models have been proposed, given that integrated care leads to better disease management, reduced symptom burden, and improved HRQoL [8]. Thus, hospitals should hold monthly multidisciplinary case conferences involving neurologists, physiotherapists, speech therapists, dietitians, mental health specialists, and social workers. Hospitals should also employ case managers and nurses, precisely assigned to PD patients, to oversee care plans and follow-up, in addition to deploying EHR systems for adequate follow-up with built-in alerts for missed appointments, unaddressed symptoms, and flagged HRQoL scores [18].

Fifth, in recommending public health awareness and early detection initiatives, it is noteworthy that early diagnosis allows for timely intervention before a significant functional or quality-of-life decline occurs [10]. Through multi-platform campaigns, statewide PD awareness campaigns by health ministries should educate the public and healthcare providers on early PD symptoms. Primary care centers should also offer free PD screening events for adults over 60 showing early motor or non-motor signs. Mandatory continuing medical education (CME) for providers to recognize early PD signs should be another measure incorporated into government and hospital policies [2].

Finally, there is a need to tackle the socioeconomic barriers and the critical role of social work. PD progression drastically alters a patient’s social determinants of health (SDOH), including income stability, housing, transportation, food access, and social connectedness, thereby compounding HRQoL decline [12,17,18]. Motor and cognitive symptoms reduce work capacity, often leading to job loss and financial instability, and consequently, housing instability. Contributing to housing instability are fall risk and disability, which make many homes unsuitable, necessitating costly home modifications. Impaired mobility and cognitive alterations create barriers to attending appointments or handling prescriptions, thus diminishing healthcare access. Further, disease progression frequently results in isolation from society and social networks. A social worker can assist by navigating resources, helping patients access disability benefits (SSDI/SSI), medication assistance programs, transportation services, food pantries, connecting patients to state-funded home modification programs or community volunteer services, facilitating enrollment in peer support groups, caregiver education programs, or telephone reassurance services for isolated patients, initiating early conversations about advance directives and future care preferences, especially while cognitive capacity is intact. Providing income and employment counseling (unemployment benefits, medical leave, vocational rehab), facilitating mental health referral by providing counseling for adjustment and caregiver stress, and linking to community or tele-mental health providers. Proactive intervention enables patients to remain engaged in decision-making, therefore mitigating potential crises such as homelessness, starvation, or institutionalization. It also allows for proactive linkage to long-term care resources, reducing preventable ER visits and hospitalizations [8,14]. Social work interventions are crucial within the whole system and essential for addressing social determinants of health obstacles and averting subsequent crises, especially when applied early in the illness trajectory.

Limitations of the study

This systematic review has several limitations. For instance, despite the pre-registration of the search strategies, no peer review was conducted. Additionally, our evidence base has been limited to only guidelines published in English, with a restriction to guidelines published between 2010 and 2025. This likely resulted in the exclusion of several pertinent published guidelines. Regardless of using pre-defined criteria and several strategies, the dependence on MeSH terms in identifying pertinent guidelines is perceived as a limitation, given that it might have omitted various relevant and applicable guidelines. Lastly, the study selection criteria also excluded non-research sources, including narrative reviews and expert opinions regarding managing elevated BP within the inpatient contexts.

## Conclusions

This systematic review affirms that Health-Related Quality of Life (HRQoL) in Parkinson’s disease (PD) is a multifaceted and multidimensional construct considerably influenced by motor and non-motor symptoms, disease progression, demographic factors, broader social determinants of health, and psychiatric comorbidities. Though motor impairments have conventionally been at the core of PD management, the various NMS symptoms have equally emerged as significant determinants of HRQoL. The NMS symptoms not only worsen disability but have been acknowledged to predict the risk of falls, minimize independence, and increase hospitalization rates in PD. Consequently, the study has disclosed that in younger-onset PD (YOPD), various unique challenges emerge as a result of the disruption of productive years, career development, and family roles, thereby increasing the psychosocial burden. Regardless of indicating increased resilience, YOPD patients normally experience protracted disease duration marked by earlier onset of complications, necessitating individualized interventions. Despite the availability of device-assisted treatments, including deep brain stimulation (DBS), as well as pharmacological treatments that proffer substantial advantages in enhancing mobility and minimizing the symptom burden, several patients are still underserved as a result of numerous access barriers, fragmentation of care models, and underdiagnosis of the non-motor symptoms. Such challenges necessitate urgent and holistic multidisciplinary interventions integrating neurological care with social work, mental health services, proactive social determinants of health interventions, and community-based rehabilitation. Evidence-based interventions, including regular NMS screening, fall prevention initiatives, integrated mental health services, system-level case coordination, and public health campaigns, may jointly enhance the HRQoL for PD patients. Further, tackling the different socioeconomic challenges that PD patients face, especially the challenges linked to housing, employment, social isolation, and transportation, is essential to reducing preventable health emergencies and sustaining long-term well-being. Lastly, enhancing HRQoL in PD not only needs symptom management but additionally a paradigm shift geared toward the provision of person-centered care, acknowledging lived experiences, psychological resilience, and social contexts of PD patients and affected persons. Healthcare systems, policymakers, and communities need to work together to effectively execute these proposed integrated interventions, ensuring that all PD patients access comprehensive, compassionate, and continuous care during their illness.
